# Spatial distribution and determinants of fertility preferences among female adolescents and young adults in Ethiopia

**DOI:** 10.1371/journal.pone.0340093

**Published:** 2026-01-06

**Authors:** Melaku Birhanu Alemu, Zohra S. Lassi, Samrawit Birhanu Alemu, Ammar Bishaw Ebrahim, Daniel Gashaneh Belay, Gizachew A. Tessema

**Affiliations:** 1 Department of Health Systems and Policy, Institute of Public Health, University of Gondar, Gondar, Ethiopia; 2 Faculty of Health Sciences, Curtin School of Population Health, Curtin University, Bentley, Western Australia, Australia; 3 Faculty of Health and Medical Sciences, Robinson Research Institute, University of Adelaide, Adelaide, South Australia, Australia; 4 Faculty of Health and Medical Sciences, School of Public Health, University of Adelaide, Adelaide, South Australia, Australia; 5 Department of Public Health, Debre Markos University, Debre Markos, Amhara, Ethiopia; 6 District Health Office, Pawi, Benishangul-Gumuz, Ethiopia; 7 Department of Epidemiology and Biostatistics, Institute of Public Health, College of Medicine and Health Sciences, University of Gondar, Gondar, Ethiopia; 8 enAble Institute, Curtin University, Bentley, Western Australia, Australia; 9 Institute for Health Research, University of Notre Dame, Fremantle, Australia; Debre Tabor University, ETHIOPIA

## Abstract

**Introduction:**

Understanding adolescents and young adults’ (AYAs) fertility preferences is crucial for guiding youth-focused family planning and predicting future fertility. However, fertility preference determinants and spatial patterns among Ethiopian AYAs remain underexplored. This study examines the spatial distribution and determinants of fertility preferences among female AYAs in Ethiopia.

**Methods:**

We analysed the nationally representative 2016 Ethiopian Demographic and Health Survey, the most recent standard survey containing fertility preference data. Fertility preference was defined as the ideal number of children respondents would like to have over their lifetime. Negative binomial regression was used to assess associations between fertility preference and sociodemographic factors, while Bayesian model-based geostatistics was employed to predict the spatial distribution of fertility preferences. Sampling weights were applied to account for the complex survey design.

**Results:**

A total of 5762 females aged 15−24 years were included in the analysis. The mean preferred ideal number of children among AYAs was 3.68 (95% confidence interval: 3.63, 3.74). Primary education (IRR: 0.91), higher education (Incidence Rate Ratio (IRR): 0.92), being unmarried (IRR: 0.86), and residing in large central areas (IRR: 0.90) were associated with a preference for fewer children. Being Muslim (IRR: 1.13), protestant (IRR: 1.17) and rural resident (IRR: 1.06) were associated with preference for more children. There was a significant spatial variation in fertility preferences. Respondents who resided in Somalia, Afar, and the western part of Gambella were found to have higher fertility preferences. The spatial regression analysis identified travel to a city (0.14, 95% Credible Interval (CI): 0.10, 0.18), urbanisation (−0.08 CI: −0.11, −0.05), income (−0.05; CI: −0.06, −0.04), and literacy (−0.06, CI −0.02, −0.01) were significant raster level predictors.

**Conclusion:**

Fertility preferences among female AYAs varied by sociodemographic characteristics, with Somali, Afar, and western Gambella regions showing higher preferences. These findings emphasise the need for context-specific policies and programs that address regional, cultural, and educational disparities. Targeted interventions in rural and underserved regions, coupled with initiatives to enhance education, empower young women, are essential to support informed and autonomous reproductive decision-making among AYAs.

## Introduction

Fertility refers to the ability of individuals or couples to bear children. It plays a significant role in shaping the size and composition of a population over time. High population growth in resource-limited settings could pose a significant challenge to sustainable development [1]. The United Nations reported that the African population could reach 2.5 billion, one in five of the world population, by 2050 [2]. Sub-Saharan Africa (SSA) accounts for more than 50% of the global fertility, with an average fertility rate of 4.8 children per woman [3,4].

High fertility is associated with rapid population growth and increased maternal and child mortality and morbidity [5]. However, fertility is perceived as highly valuable and desirable in Sub-Saharan Africa (SSA), which is more prominent among young women [6]. Parents in low socio-economic status desire more children to have support in farming and emotional, and economic support in their old age [7]. Consequently, fertility and population growth are much higher than in other parts of the world [8]. Among the sub-Saharan African countries, Ethiopia is one of the developing countries with high fertility and rapid population growth [9].

Preferred family size is defined as the number of children wanted in one’s lifetime [10]. The ideal number of children of a woman is a critical measure for predicting high-risk fertility behaviour [11]. Studies identified that the actual number of children women has influenced their preferred number of reported children. African studies have recorded considerable changes in women’s fertility preferences over time [12,13].

The adolescent childbirth rate was 4.4 births per 1000 women in SSA, which was significantly higher compared to a global average of 1.5 births per 1000 women [14]. In low and middle-income countries (LMICs), almost 21 million adolescent girls become pregnant every year [14]. According to the EDHS 2016 report, the total fertility rate in Ethiopia was 4.6 children per woman [15]. In Ethiopia, the high fertility rate has been contributing to the unprecedented increases in maternal and child mortality [16].

Adolescents are at a higher risk of pregnancy-related morbidity and mortality [17]. In LMICs, a large number of girls become pregnant, of which approximately 5.6 million women have abortions, which leads to morbidity and mortality [14]. Over 800 women died every day all over the world from preventable maternal and childbirth-related causes in 2020. Almost 95% of deaths occurred in low and lower-middle-income countries [18]. The third Sustainable Development Goal (SDG-3) will continue to prioritise preventing preventable maternal and child deaths, bringing down neonatal mortality to 12 or fewer deaths per 1,000 live births and worldwide maternal mortality to fewer than 70 deaths per 100,000 live births [19]. However, Ethiopia has the high maternal mortality ratio with 412 deaths per 100,000 live births [15]. According to the Demographic and Health Survey, neonatal mortality increased from 29 in 2016–33 in 2019 fatalities per 1,000 live births [7].

According to various studies, factors at individual and community levels, such as age, education, marital status, religion, occupation, region, parity, residence, and wealth, are associated with fertility preference [20–22]. Although fertility preference in the general population has been studied in the past [23,24], no research has explicitly looked at the factors and spatial variation among female AYAs in Ethiopia. Understanding AYAs’ fertility preferences is key to anticipating reproductive behaviour and guiding youth-focused family planning [25]. Thus, the present study aims to identify the determinants associated with the ideal number of children in Ethiopia, considering the socioeconomic, demographic and health-related determinants that influence the ideal number of children among Ethiopian AYAs. The study also aims to assess the geographical variation of an ideal number of children preferred among AYAs in Ethiopia. This helps to design geographically focused interventions in Ethiopia. This study could help healthcare planners and policymakers to design evidence-based interventions and the appropriate allocation of resources in areas with high fertility preference.

## Methods

### Study settings and data source, and study population

This study utilised secondary data from the 2016 Ethiopian Demographic and Health Survey (EDHS), which was a community-based cross-sectional survey conducted in Ethiopia from 18 January to 27 June 2016. Ethiopia is in East Africa and borders Eritrea to the north, Sudan and South Sudan to the west, Somalia and Djibouti to the east, and Kenya to the south. Ethiopia’s population is estimated to be 126 million people, with approximately 29 million women of reproductive age [26]. The EDHS data utilised in this study are publicly accessible via the Demographic and Health Surveys program website (https://dhsprogram.com/). The authors obtained permission to use the dataset on 19 July 2023. All data were fully anonymised, and no access to individual identifiers was granted.

### Sample procedure and sample size

The 2016 EDHS sample used a multi-stage stratified sampling technique with two stages. Each region was stratified into urban and rural areas. In the first stage, 645 EAs were selected with a probability method proportional to the enumeration area size by independent selection in each sampling stratum. In the second selection stage, a fixed number of 28 households per cluster were selected with an equal probability, systematic sampling from the newly created household listings. A detailed sampling procedure can be found in another report [27]. Of 15,683 women who completed the individual interviews, we included 5,762 adolescents and young adult women (15–24 years) for the analysis.

### Measure of variables

#### Outcome variable.

Fertility preference was the outcome variables, defined as the specific number of children a respondent would choose to have over their lifetime if they could return to a time before having any children and make this choice freely [28]. This measure is a common indicator of fertility preference and used to predict future reproductive behaviour and fertility-related decision-making [15,29].

#### Independent variable.

Age (coded as adolescents (15 –19 ) and young adults (20 –24 )), maternal occupation (coded as “working” and “Not working”), women’s education (coded as “no education”, “primary: and “secondary and above”), marital status (recoded as “currently married” (married/ live with a partner) and “unmarried” (single/divorced/separated/widowed)), wealth index (categorised as “poorest”, “poor”, “middle”, “rich”, and “richest”), household head sex (coded as Male and Female), religion (coded as Orthodox, Muslim, Protestant and Others), family size (coded as small (1 –4 ) and High (> 4)), media access (coded as Yes and No), substance use (No substance and substance used) and residence (coded as urban and rural). The region was recorded as “Large central”(Tigray, Amhara, Oromia, SNNP), “Small peripheral” (Afar, Gambella, Harari, Somali) and “Metropolises” (Addis Ababa and Dire Dawa).

### Statistical analysis

The data were weighted using sampling weights and strata to restore the survey’s representativeness. Missing values were handled and cleaned before any statistical analysis. Non-numeric responses were excluded from the analysis. Respondents who prefer to have more than 16 children were excluded from this study as having more than 16 children in a lifetime is not common [22]. Mean and percentage were conducted to describe participants’ socioeconomic and demographic characteristics and presented with a frequency percentage table. STATA 17 was used to conduct the regression analysis.

Since the outcome variable (i.e., ideal number of children) was the count variable, we considered the count data regression model. A count variable is a variable that takes on discrete values reflecting the number of occurrences of an event in a fixed time. The best regression model for the count data was chosen by checking for over-dispersion and zero problems of the data distributions. Over-dispersion can be tested by either comparing the mean and variance for differences or checking the ratio of deviance to degrees of freedom, if it is around 1. The mean and variance of fertility preferences were 3.68 and 4.54 children, respectively, indicating over-dispersion in our data. Thus, negative binomial regression fits the data appropriately as it accounts for the over-dispersion of data [30,31]. Additionally, it provides more accurate standard errors and p-values than the basic Poisson regression [30,32]. Furthermore, we have checked for zero problems by plotting a density distribution using a histogram for the data. However, our data have no excessive zero problem, indicating that a negative binomial model is appropriate for our data.

We have estimated the incidence rate ratios of the explanatory variables. Furthermore, we have estimated the predicted fertility preferences for explanatory variables and their interaction with other variables.

The spatial analysis was conducted using the geographic information system (GIS) of the 2016 EDHS to map fertility preference distribution and predict the spatial dependence using raster data. The ideal number of children preferred in each data collection cluster was calculated and linked to the GIS. R software 4.4.3 and ArcGIS version 10.7 were used for spatial analysis and visualisation, respectively.

The geographic distribution of the ideal number of children for AYAs at the cluster level is presented. A high number of child preferences was represented by a red dot, while a low number of child preferences was represented by a blue dot.

The hot spot analysis was conducted using the Getis-Ord Gi* analysis. It identifies areas with the high ideal number of children (hotspots) and with the low ideal number of children (cold spots). A positive Z-score with p < 0.05 indicates a significant hotspot, while a negative Z-score with p < 0.05 indicates a significant cold spot (S1 Supplementary).

#### Model-based geospatial analysis.

We employed a Bayesian model-based geostatistical approach to estimate the spatial distribution of fertility preference [33], which is used for better spatial mapping and prediction [34]. Travel time to the nearest city, income, literacy, and urban residence [35,36] were included as a covariate. Due to the count nature of the outcome and overdispersion, we specified a Negative Binomial regression model with a log link function. Inference was conducted using R-INLA and the Stochastic Partial Differential Equations (SPDE) approach, enabling efficient modelling of spatial autocorrelation [33,37]. The model generated high-resolution (1 km²) maps and posterior summaries, including means, standard deviations, and 95% credible intervals for fertility preference were reported.

#### Ethics approval.

The Ethiopian demographic health survey (DHS) dataset is available to the public upon request from the DHS website https://dhsprogram.com/. We submitted a request to the DHS by briefly stating the objectives of this analysis and thereafter received permission to download the maternal record dataset.

## Results

### Socio-demographic characteristics

A total of 5762 women were included in the analysis. Young adults (20–25 years) were lower (45%) than adolescents aged 15–19. The majority (54.8%) of the respondents had a primary education. Most of the respondents’ religion was Orthodox (44%), followed by Muslims (30%). Around three in four respondents were from rural parts of Ethiopia. Likewise, most participants (88%) live in large and central regions. Furthermore, most participants reside in households with more than four family members (Table 1).

**Table 1:** Socio-demographic characteristics of adolescent and young women aged 15-49 in Ethiopia, EDHS 2016 (n = 5762).

Variables	Category	Frequency	Percentage (%)
Age	Adolescents (15 –19 )	3177	55.1
	Young adults (20 –24 )	2585	44.9
Household Head Sex	Male	4340	75.3
	Female	1422	24.7
Maternal Education	No education	1065	18.5
	Primary	3159	54.8
	Secondary and above	1538	26.7
Marital status	Currently Married	2038	35.4
	Unmarried	3724	64.6
Religion	Orthodox	2516	43.7
	Muslim	1701	29.5
	Protestant	1440	25.0
	Others*	105	1.8
Occupation	Not working	3234	56.1
	Working	2528	43.9
Residence	Urban	1426	24.7
	Rural	4336	75.3
Region	Metropolis	446	7.7
	Large centrals	5054	87.7
	Small peripherals	263	4.6
Wealth	Poorest	885	15.4
	Poorer	1081	18.8
	Middle	1210	21.0
	Richer	1239	21.5
	Richest	1347	23.4
Substance use	No substance	5271	91.5
	Substance used	491	8.5
Family size (current)	Small (1 –4 )	2,490	43.2
	High (>4)	3,272	56.8
Number of children	0	4079	70.8
	1	1012	17.6
	2 and more	671	11.6
Media Access	Yes	2993	52.0
	No	2769	48.0

* Traditional, catholic, other religions.

### The ideal number of children

**Fig 1:**
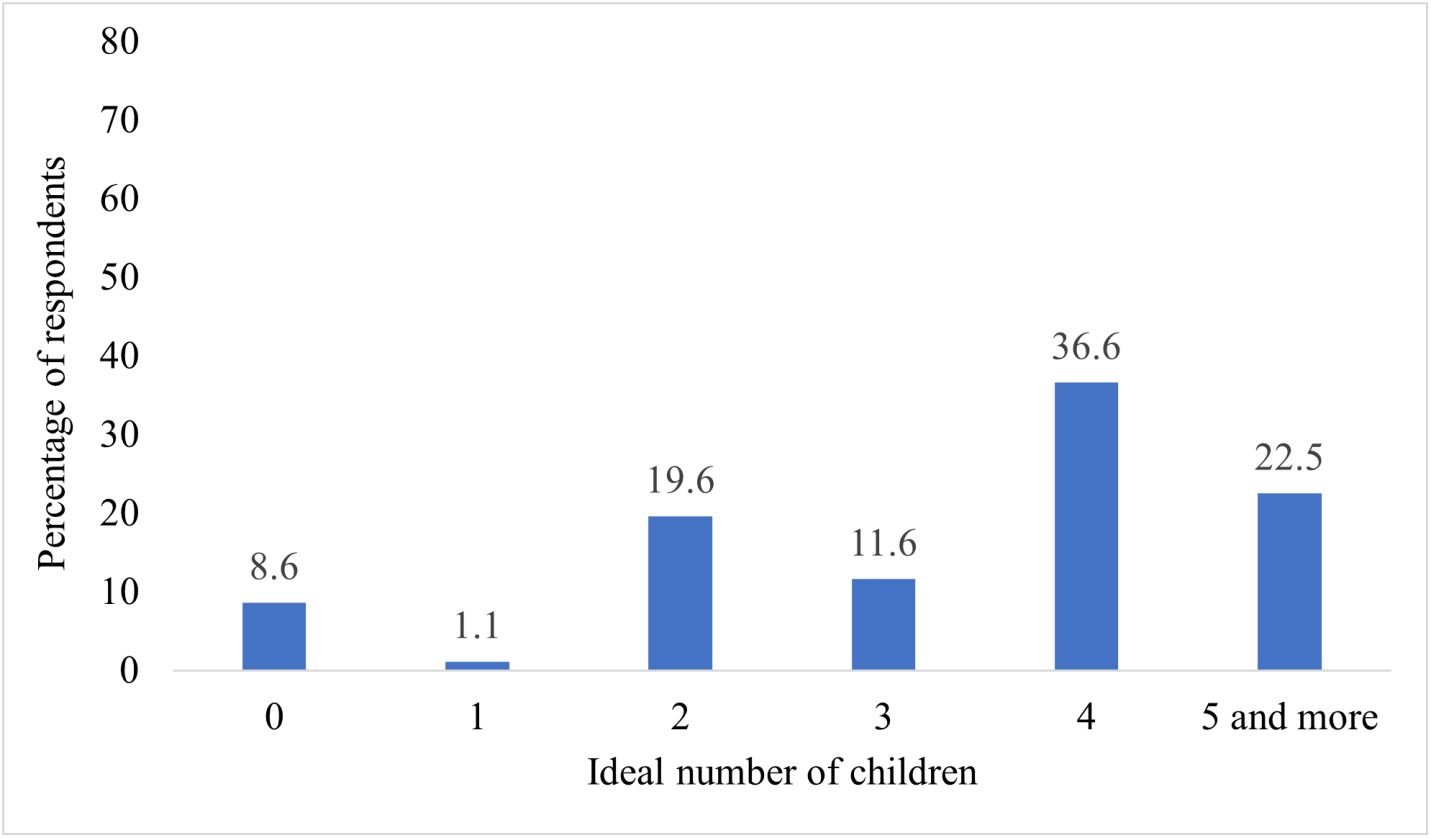
Percent distribution of fertility preferences among female adolescents and young adults in Ethiopia, EDHS 2016.

The ideal number of children was recoded to 16 categories in the dataset (0–15) after excluding unrealistic preferences. The data consisted of the ideal number of children preference of AYAs aged 15–24, with no child being the worst possible outcome. Around one-third (37%) of AYAs preferred to have four children. The mean ideal number of children preference of the woman was 3.68 (95% CI: 3.63, 3.74), with a variance of 4.54 in Ethiopia (Fig 1).

### Determinants of the ideal number of children

We found that education, marital status, religion, residence and region had significant incidence risk ratios. When all other variables were held constant, the number of ideal children for women with secondary and higher education and primary education was 0.92 (IRR = 0.92, 95% CI: 0.85, 0.99) and 0.91 (IRR = 0.91, 95% CI: 0.85, 0.97) times less, respectively. The number of ideal children for not-married women is 0.86 times (IRR = 0.86, 95% CI: 0.81, 0.91) less than that of married women. The number of ideal children for Muslim and Protestant women was 1.13 (IRR = 1.13, 95% CI: 1.06, 1.2) and 1.17 (IRR = 1.17, 95% CI: 1.12, 1.23) times higher, respectively. Compared to urban residents, rural residents’ ideal number of children is 1.06 times (IRR = 1.06, 95% CI: 1.01, 1.12). Similarly, the ideal number of children for women residing in larger central regions is 0.90 times (IRR = 0.90, 95% CI: 0.85, 0.95) lower than that in metropolis regions. However, the ideal number of children for women residing in smaller peripheral regions is 1.59 times (IRR = 1.59, 95% CI: 1.47, 1.71) higher than those residing in metropolis regions (Table 2).

**Table 2:** Predictors of ideal number of children preferred among adolescent and young women (15 –24 ) in Ethiopia, EDHS 2016 (n = 5762).

Variable	Categories	IRR	Std. err.	95% CI for IRR	
				Lower	Upper
Age	15-19	Ref			
	20-24	1.02	0.02	0.97	1.07
Education	No education	Ref			
	Primary	0.91**	0.03	0.85	0.97
	Secondary & above	0.92*	0.04	0.85	0.99
Marital status	Married	Ref			
	Not married	0.86***	0.02	0.81	0.91
Household sex	Male	Ref			
	Female	1.01	0.03	0.96	1.07
Religion	Orthodox	Ref			
	Muslim	1.13***	0.03	1.06	1.20
	Protestant	1.17***	0.03	1.12	1.23
	Others^a^	1.21	0.17	0.92	1.58
Occupation	Not working	Ref			
	Working	1.03	0.02	0.98	1.07
Family size	<4	Ref			
	>4	1.03	0.02	0.98	1.07
Media	No	Ref			
	Yes	1.00	0.02	0.95	1.04
Substance	No substance	Ref			
	Substance used	1.02	0.05	0.92	1.12
Wealth	Poorest	Ref			
	Poorer	0.97	0.04	0.90	1.05
	Middle	0.93	0.03	0.87	1.003
	Richer	0.95	0.04	0.88	1.02
	Richest	0.93	0.04	0.86	1.001
Residence	Urban	Ref			
	Rural	1.06*	0.03	1.01	1.12
Region	Metropolises	Ref			
	Large centrals	0.90***	0.03	0.85	0.95
	Small peripherals	1.59***	0.06	1.47	1.71
_cons		4.13***	0.21	3.74	4.57
Ln alpha		−4.53	0.83	−6.16	−2.9
alpha		0.01	0.01	0.002	0.055

^a^Traditional, catholic, other religions; *P value < 0.01; **P value < 0.05; ***P value < 0.001.

A separate regression model was conducted for small peripheral areas to understand the predictors responsible for higher fertility preferences (S2 Supplementary).

There were significant interactions between predictors for the predicted number of children preferred. A significant interaction was found between religion with age, family size with age, wealth with age, substance use with education, wealth with education, religion with marital status, family size with marital status, media exposure with marital status, wealth with marital status, family size with religion, substance use with religion, region with religion, substance use with occupation, wealth with family size, residence with family size and region with family size. The estimates of the interaction are presented in the S3 Supplementary.

Family size has significantly increased the predicted number of fertility preferences among young adults (aged 20–24). However, family size does not significantly impact adolescent fertility preferences (aged 15–19). Adolescent respondents from the poorest households significantly prefer children to those from richer households. However, wealth status has no significant contribution to young adults. Muslim respondents who live in small peripheries have significantly higher fertility preferences. However, Muslim respondents who live in large central regions had preferences like those of other regions (S4 Supplementary).

### Spatial distribution

**Fig 2:**
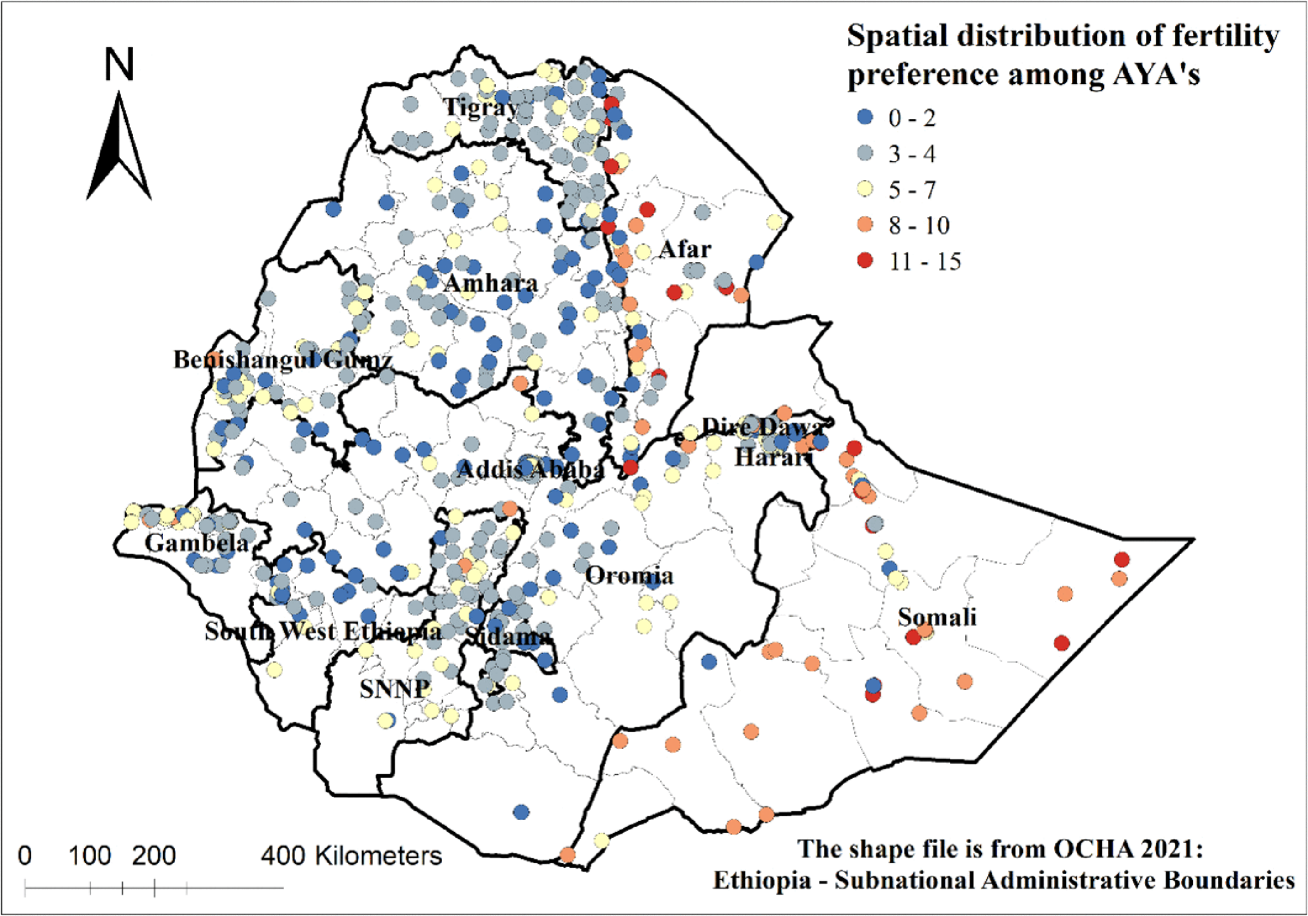
Spatial distribution of fertility preferences among female adolescents and young adults in Ethiopia, EDHS 2016.

The spatial distribution of fertility preferences among adolescents and young adults in Ethiopia was illustrated below. Areas marked with deep blue dots indicate a preference for 0–2 children, while deep red areas represent a preference for 11–15 children (Fig 2).

### Spatial prediction of high fertility preference

**Fig 3:**
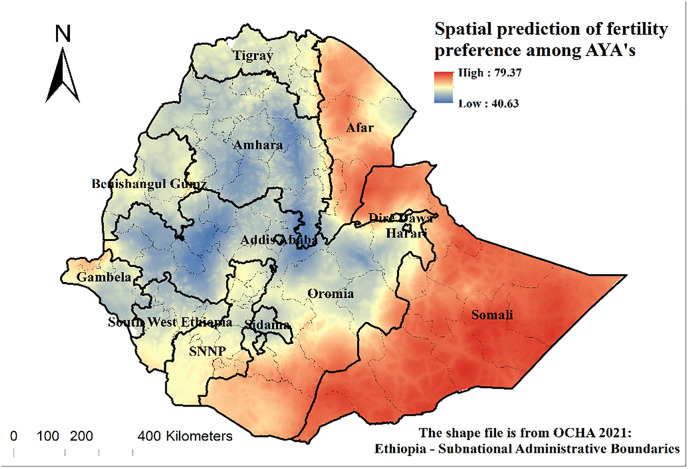
Spatial prediction of fertility preferences among female adolescents and young adults in Ethiopia, EDHS 2016.

The spatial prediction of high fertility preference showed a high fertility preference mainly in the Somali region, followed by the Afar, northern Gambella and the southern part of Southwest Ethiopia, and south and east of the Oromia region. There are low preferences for fertility in the Amhara and western parts of the Oromia regions, as well as in Sidama, Dire Dawa city administration, and Addis Ababa city administration. The predicted high fertility preference is shown in red colour (Fig 3).

### Factors associated with the spatial distribution of fertility preference

The spatial regression analysis identified key factors associated with fertility preference in Ethiopia. Travel to a city, urban, income, and literacy were significant predictors. Higher income was significantly associated with lower fertility preference (−0.051; 95% Credible Interval: −0.061, −0.041), indicating that women with greater income levels tend to prefer fewer children (Table 3).

**Table 3:** Factors associated with the spatial distribution of fertility preference in Ethiopia.

Variable	Mean (95% Credible Interval)
Travel to a city	0.135 (0.096, 0.175) *
Urban	−0.077 (−0.107, −0.048) *
Income	−0.051 (−0.061, −0.041) *
Literacy	−0.015 (−0.022, −0.008) *
Intercept	0.751 (0.735, 0.767) *

* Significant at P-value < 0.05.

## Discussion

This study assesses determinants of an ideal number of children’s preferences and the pattern of spatial variation among AYAs in Ethiopia. This study found that education, marital status, religion, and rural residence were key determinants of fertility preference among AYAs in Ethiopia. Additionally, notable geographic variation was observed, with higher fertility preferences concentrated in Somali, Afar, and Gambella regions.

The mean ideal number of children preferred for a woman was 3.68 in Ethiopia. The figure is slightly lower than the average number of children a woman has in Ethiopia which was 4.6 [38]. The difference could be arisen from partner preference, unplanned pregnancy, change in preference over time, child gender preference. The fertility preference of Ethiopia is higher than a study in Indonesia [39], which was 2.7 in 2017 which was conducted in the same age group. This could be due to Indonesians’ recent years’ delayed marriages and intention not to have a child poses a significant decrease in childbearing desire [40]. However, the mean number of children preferred is lower than in studies done in sub-Saharan Africa [41] and in Ethiopia [24,42]. The variation might be due to cultural differences between Ethiopia and other Sub-Saharan African countries, which have a higher preference for children because of support in farming and finance in their old age [7]. However, the variation between studies done in Ethiopia might be due to the difference in the source population. Other studies used women aged 15–49 as a source population, which increased the mean number of children preferred. A study identified that being in a higher age group is associated with a higher number of children’s preferences [43].

Furthermore, women’s education level is inversely related to the ideal number of children. As the educational level of women increases, women’s preference for more children decreases. This finding is consistent with a study conducted in America [44], in Ethiopia [24,45] and in Uganda [46]. The result is logical, as uneducated women may not understand family limitations; rather, they consider their children an asset. Furthermore, less educated women are expected to be unemployed, which results in spending most of their time as housewives, which again makes them comfortable having more children still [7,42]. This implies that education had a significant role in women’s fertility preferences.

Marital status and religion were found to be the determinants of fertility preference. The study found that adolescents who were married were more likely to prefer a larger number of children than those who were never married. This result was consistent with studies done in Sub-Saharan countries [41] and Nigeria [47]. Muslim and protestant religious followers have a significantly higher fertility preference when compared to Orthodox followers. The possible reasons could be that different religious followers have different beliefs about reproductive behaviour and the value of having children. Different studies also reported the association between religion and high fertility preference [24,43,48,49].

Region and residence were significantly associated with more children’s preferences. Adolescent and young women in small peripheral and rural regions had higher fertility preferences than in metropolises and urban areas. This result is consistent with other studies, which stated regional differences in the ideal number of children’s preference [24,47]. This may be because women who live in metropolises and urban areas are more likely to be educated and have access to media and fertility and family planning information. Education and media access in urban areas increase access and exposure to reproductive information and the importance of family planning in limiting family size, which will decrease fertility preference among women [22]. Furthermore, in the pastoralist communities, having many children is seen as a source of wealth, labour, social status, and future security, deeply rooted in cultural, economic, and religious values [50]. Various studies also indicate that this region had higher odds of higher fertility preference [45,48].

The spatial distribution of fertility preference significantly differed in different areas in Ethiopia. This finding was evidenced by a study conducted in Nigeria [47] and Ethiopia [24]. Afar, Somali and the western part of Gambella had high fertility preferences among AYAs. This might be due to large number of peoples from those regions are rural residents, where cultural beliefs, economic status and access to health services affect ideal number of children [51,52]. In rural areas, insufficient number of health professionals, a lack of infrastructure, roads and transportation can also be a barrier toward receiving awareness on family planning as well as professional medical care [53]. Additionally, in some areas of those regions, cultural and regional beliefs of having large families seen as a blessing [53]. Furthermore, Families in poverty, particularly those who make their living through agriculture, may have more kids as a way of supporting the family’s livelihood [54,55]. A study conducted using all reproductive age groups found spatial clustering only in the Somali region [24]. This implies that AYAs have distinct fertility preferences from reproductive-aged women, and special attention should be given to AYAS women in Afar and the western part of Gambella. Furthermore, Income status significantly affected the spatial distribution of fertility preferences. AYAS from high-income areas had lower fertility preferences than AYAS from low-income areas.

Different studies also found factors like male partner attitude towards fertility and contraceptive use to be associated with the difference in fertility preference across different regions in Ethiopia [56,57]. In regions like Afar where men hold more conservative fertility ideals or disapprove of modern contraception, women are less likely to report limiting or spacing intentions [58]. The possible reasons for this might be men are mostly the head of house hold who makes a decision and men in rural and peripheral regions are less likely to be educated and access less information about reproductive health benefits of limiting children [59,60]. Addition to this, child gender preference could be one factor that contribute in regional variation [61]. Different regions gender norms, with stronger son preference reported in some cultural and religious groups, which might contribute for persistent high fertility desires observed in different regions [61–63].

### Strengths and limitations of the study

A key strength of this study is its focus on AYAs, which reduces bias from prior childbearing in reporting fertility preferences, especially since nearly 70% of respondents had no children. We also accounted for the count nature of the outcome using negative binomial regression, which helps reduce classification bias. Additionally, we showed the interaction of sociodemographic variables with fertility preferences. Furthermore, the use of Bayesian model-based geostatistics enabled high-resolution spatial estimates and the identification of spatial disparities. However, the study has several limitations. Fertility preference, measured by the ideal number of children, may still be influenced by existing childbearing. The cross-sectional design limits the ability to draw causal conclusions, and responses may be affected by social desirability bias. While our analysis was based on the most recent available data on fertility preferences (collected in 2016), it is important to acknowledge that societal changes such as greater media exposure, higher educational attainment, and increased cost of living may have influenced fertility attitudes and behaviours in the last decade. Predictors such as marital status and educational attainment may be endogenous, as fertility preference can influence these factors. Finally, the study reflects only women’s preferences, even though fertility decisions are also shaped by partners, the gender of existing children, access to contraception, and broader social and cultural influences.

## Conclusion

Fertility preferences of female AYAs were significantly influenced by sociodemographic characteristics. Education, marital status, religion, and geographic location were crucial in shaping fertility preferences. Furthermore, the spatial variation of female AYAs was different from the fertility preferences of reproductive-aged women. Female AYAs in Somali, Afar, and western Gambella regions showed a stronger preference for larger families. These findings suggest that fertility preferences were shaped by individual characteristics and cultural, religious, and regional factors, emphasising the importance of designing reproductive health policies that address these varied individual and community-level factors. Policy makers and stakeholders should design programs aimed at promoting reproductive health and family planning particularly in rural areas and regions like Afar and Somali. Addressing socio-economic and educational disparities, can contribute to informed reproductive choices among adolescent and young women.

## Supporting information

S1 Supplementary Hotspot analysis of fertility preferences among adolescents and young adults. (TIF)

S2 Supplementary Predictors of ideal number of children among female adolescents and young adults, Ethiopia. Negative binomial regression analysis of predictors associated with fertility preferences among female adolescents and young adults in small peripheral regions of Ethiopia, using EDHS 2016 data (n = 263).
(DOCX)

S3 Supplementary Analysis of the interaction effect of sociodemographic variables. (DOCX)

S4 Supplementary Interaction effect of variables on fertility preference. (DOCX)
