# Supplementary material for: Spatial distribution and determinants of fertility preferences among female adolescents and young adults in Ethiopia
Source: PLoS One. 2026 Jan 6;21(1):e0340093. doi: 10.1371/journal.pone.0340093 (PMC12774340; doi:10.1371/journal.pone.0340093)
Supplement: S2 Supplementary — Negative binomial regression analysis of predictors associated with fertility preferences among female adolescents and young adults in small peripheral regions of Ethiopia, using EDHS 2016 data (n = 263). (DOCX) [file pone.0340093.s002.docx]

S2 Supplementary: Predictors associated with the ideal number of children preferred among adolescent and young women (15-24) among small peripheral regions in Ethiopia, EDHS 2016 (n=263): Negative Binomial regression analysis

| Variable | Categories | IRR | Std. err. | 95% CI for IRR |
| --- | --- | --- | --- | --- |
|  |  |  |  | Lower **-** Upper |
| Age | 15–19 | Ref |  |  |
|  | 20–24 | 0.97 | 0.05 | 0.87 – 1.09 |
| Education | No education | Ref |  |  |
|  | Primary | 0.95 | 0.06 | 0.85 – 1.07 |
|  | Secondary & above | 0.91 | 0.08 | 0.77 – 1.09 |
| Marital status | Married | Ref |  |  |
|  | Not married | 0.94 | 0.06 | 0.83 – 1.06 |
| Household sex | Male | Ref |  |  |
|  | Female | 1.02 | 0.05 | 0.93 – 1.13 |
| Religion | Orthodox (Ref) | Ref |  |  |
|  | Muslim | 1.94*** | 0.20 | 1.59 – 2.38 |
|  | Protestant | 1.09 | 0.15 | 0.84 – 1.43 |
|  | Others ^a^ | 1.56 | 0.41 | 0.93 – 2.62 |
| Occupation | Not working | Ref |  |  |
|  | Working | 0.90 | 0.05 | 0.80 – 1.01 |
| Family size | <4 | Ref |  |  |
|  | >4 | 0.99 | 0.05 | 0.89 – 1.10 |
| Media | No | Ref |  |  |
|  | Yes | 0.99 | 0.06 | 0.88 – 1.12 |
| Substance use | No substance | Ref |  |  |
|  | Substance used | 0.80 | 0.12 | 0.60 – 1.07 |
| Wealth | Poorest | Ref |  |  |
|  | Poorer | 1.00 | 0.07 | 0.87 – 1.15 |
|  | Middle | 0.96 | 0.08 | 0.81 – 1.14 |
|  | Richer | 0.95 | 0.09 | 0.79 – 1.13 |
|  | Richest | 0.84 | 0.09 | 0.68 – 1.03 |
| Residence | Urban | Ref |  |  |
|  | Rural | 1.06 | 0.07 | 0.93 – 1.21 |
| Constant |  | 4.26*** | 0.56 | 3.30 – 5.52 |
| ^a^ Traditional, wakefeta, non-believer; * P value < 0.05 | | | | |
