# Supplementary material for: Spatial distribution and determinants of fertility preferences among female adolescents and young adults in Ethiopia
Source: PLoS One. 2026 Jan 6;21(1):e0340093. doi: 10.1371/journal.pone.0340093 (PMC12774340; doi:10.1371/journal.pone.0340093)
Supplement: S3 Supplementary — (DOCX) [file pone.0340093.s003.docx]

S3 Supplementary: The interaction effect of sociodemographic variables for fertility preference

| Variables | Categories | IRR | Std. Err. | P Value |
| --- | --- | --- | --- | --- |
| Age | 15-19 | Ref |  |  |
|  | 20-24 | 0.82 | 0.08** | 0.047 |
| Education | No education | Ref |  |  |
|  | Primary | 0.90 | 0.14 | 0.507 |
|  | Secondary & above | 0.96 | 0.18 | 0.817 |
| Marital status | Married | Ref |  |  |
|  | Not married | 0.63 | 0.08*** | 0.000 |
| Household sex | Male | Ref |  |  |
|  | Female | 0.72 | 0.09*** | 0.006 |
| Religion | Orthodox | Ref |  |  |
|  | Muslim | 1.13 | 0.18 | 0.441 |
|  | Protestant | 0.97 | 0.13 | 0.837 |
|  | Others | 0.99 | 0.34 | 0.965 |
| Occupation | Not working | Ref |  |  |
|  | Working | 1.12 | 0.11 | 0.263 |
| Family size | <4 | Ref |  |  |
|  | >4 | 1.10 | 0.12 | 0.372 |
| Media | No | Ref |  |  |
|  | Yes | 1.02 | 0.15 | 0.875 |
| Substance | No substance | Ref |  |  |
|  | Substance used | 1.59 | 0.40* | 0.064 |
| Wealth | Poorest | Ref |  |  |
|  | Poorer | 0.98 | 0.17 | 0.905 |
|  | Middle | 0.95 | 0.15 | 0.732 |
|  | Richer | 0.72 | 0.13* | 0.067 |
|  | Richest | 0.85 | 0.16 | 0.399 |
| Residence | Urban | Ref |  |  |
|  | Rural | 1.11 | 0.17 | 0.515 |
| Region | Metropolises | Ref |  |  |
|  | Large centrals | 1.18 | 0.21 | 0.356 |
|  | Small peripherals | 1.42 | 0.26 | 0.053 |
| Religion# Age | Orthodox #15-19 | Ref |  |  |
|  | Muslim#20-24 | 1.22 | 0.09** | 0.004 |
|  | Protestant#20-24 | 1.06 | 0.06 | 0.336 |
|  | Others#20-24 | 0.55 | 0.12*** | 0.006 |
| Family size# Age | <4#15-19 | Ref |  |  |
|  | >4#20-24 | 1.12 | 0.06** | 0.022 |
| Wealth # Age | Poorest#15-19 | Ref |  |  |
|  | poorer#20-24 | 1.14 | 0.10 | 0.115 |
|  | middle#20-24 | 1.17 | 0.09** | 0.047 |
|  | richer#20-24 | 1.14 | 0.10 | 0.123 |
|  | richest#20-24 | 1.20 | 0.11** | 0.045 |
| Substance # Education | No substance# No education | Ref |  |  |
|  | Substance used# Primary | 0.86 | 0.11 | 0.223 |
|  | Substance used# Secondary & above | 0.65 | 0.13** | 0.032 |
| Wealth# Education | Poorest# No education | Ref |  |  |
|  | Poorer #Primary | 0.87 | 0.08 | 0.117 |
|  | Poorer# Secondary & above | 0.78 | 0.10** | 0.047 |
|  | Middle# Primary | 0.93 | 0.09 | 0.449 |
|  | Middle# Secondary & above | 0.90 | 0.10 | 0.332 |
|  | Richer# Primary | 1.19 | 0.13 | 0.112 |
|  | Richer# Secondary & above | 1.24 | 0.16* | 0.096 |
|  | Richest# Primary | 1.00 | 0.13 | 0.981 |
|  | Richest# Secondary & above | 0.89 | 0.12 | 0.371 |
| Religion# Marital status | Orthodox# Married | Ref |  |  |
|  | Muslim# Not married | 1.18 | 0.10** | 0.038 |
|  | Protestant# Not married | 1.17 | 0.08** | 0.016 |
|  | Others# Not married | 0.71 | 0.21 | 0.241 |
| Family size# Marital status | <4# Married | Ref |  |  |
|  | >4# Not married | 1.17 | 0.07*** | 0.008 |
| Media # Marital status | No# Married | Ref |  |  |
|  | Yes # Not married | 1.17 | 0.07** | 0.010 |
| Substance # Marital status | No substance# Married | Ref |  |  |
|  | Substance used# Not married | 0.88 | 0.11 | 0.310 |
| Wealth# Marital status | Poorest # Married | Ref |  |  |
|  | Poorer # Not married | 1.13 | 0.11 | 0.201 |
|  | Middle # Not married | 1.22 | 0.11** | 0.025 |
|  | Richer # Not married | 1.07 | 0.10 | 0.480 |
|  | Richest # Not married | 1.07 | 0.11 | 0.482 |
| Wealth# Household head sex | Poorest# Male | Ref |  |  |
|  | Poorer # Female | 1.19 | 0.10** | 0.035 |
|  | Middle # Female | 1.10 | 0.09 | 0.230 |
|  | Richer # Female | 1.13 | 0.09 | 0.135 |
|  | Richest # Female | 1.13 | 0.10 | 0.139 |
| Family size# Religion | <4#Orthodox | Ref |  |  |
|  | >4#muslim | 0.97 | 0.07 | 0.626 |
|  | >4#protestant | 1.05 | 0.06 | 0.403 |
|  | >4#others | 1.66 | 0.43** | 0.050 |
| Substance # Religion | No substance# Orthodox | Ref |  |  |
|  | Substance used# Muslim | 0.69 | 0.08*** | 0.002 |
|  | Substance used# protestant | 0.85 | 0.21 | 0.519 |
|  | Substance used# others | 0.64 | 0.23 | 0.205 |
| Region # Religion | Metropolises # Orthodox | Ref |  |  |
|  | Large centrals# Muslim | 0.88 | 0.08 | 0.130 |
|  | Large centrals# Protestant | 1.16 | 0.09* | 0.063 |
|  | Large centrals# Others | 0.83 | 0.44 | 0.725 |
|  | Small peripherals# Muslim | 1.53 | 0.13*** | 0.000 |
|  | Small peripherals# Protestant | 1.12 | 0.10 | 0.240 |
|  | Small peripherals# Others | 0.65 | 0.40 | 0.476 |
| Substance # Occupation | No substance # Not working | Ref |  |  |
|  | Substance used# Working | 1.33 | 0.13*** | 0.003 |
| Wealth # Family size | Poorest#<4 | Ref |  |  |
|  | Poorer#>4 | 0.91 | 0.07 | 0.235 |
|  | Middle#>4 | 0.80 | 0.06*** | 0.003 |
|  | Richer#>4 | 0.82 | 0.06*** | 0.006 |
|  | Richest#>4 | 0.85 | 0.07 | 0.042 |
| Residence# Family size | Urban#<4 | Ref |  |  |
|  | Rural#>4 | 1.14 | 0.07** | 0.038 |
| Region # Family size | Metropolises #<4 | Ref |  |  |
|  | Large centrals#>4 | 0.85 | 0.05** | 0.010 |
|  | Small peripherals#>4 | 0.80 | 0.07*** | 0.006 |
| Cons |  | 4.32 | 0.84*** | 0.000 |

IRR: Incidence Rate Ratio
