# Supplementary material for: Spatial distribution and determinants of fertility preferences among female adolescents and young adults in Ethiopia
Source: PLoS One. 2026 Jan 6;21(1):e0340093. doi: 10.1371/journal.pone.0340093 (PMC12774340; doi:10.1371/journal.pone.0340093)
Supplement: S4 Supplementary — (DOCX) [file pone.0340093.s004.docx]

**A**

**B**

**C**

**D**

**E**

**F**

**S4 Supplementary: Interaction effect of variables on fertility preference.**

**G**

**H**

**I**

**J**

**K**

**L**

**S4 Supplementary: Interaction effect of variables on fertility preference (continued).**

**M**

**N**

**O**

**P**

**Q**

**R**

**S4 Supplementary: Interaction effect of variables on fertility preference (continued).**
